# Training strategies for neck ultrasound examination and ultrasound-guided cervical lymph node biopsies: a narrative review

**DOI:** 10.1186/s12909-025-08496-7

**Published:** 2026-01-21

**Authors:** Lorraine Thong, Cyrus Daneshvar, Maged Hassan, Mairead Boohan, David P. Breen

**Affiliations:** 1 Interventional Respiratory Unit, Galway University Hospital, Galway, Republic of Ireland; 2 Department of Medical Education, Queen’s University Belfast, University Road, Belfast, Northern Ireland United Kingdom; 3 Department of Thoracic Medicine, University Hospitals Plymouth NHS Trust, Plymouth, United Kingdom; 4 Chest Diseases Department, Alexandria University Faculty of Medicine, Alexandria, Egypt; 5 University of Galway, University Road, Galway, Republic of Ireland

**Keywords:** Lung cancer, Training, Neck US, Cervical lymph node, Biopsy, Medical education, Simulation training, Competency-based assessment

## Abstract

Lung cancer is the leading cause of cancer-related death world-wide. Cervical/neck lymph nodes are often involved in lung cancer; however, neck ultrasound (US) examination and US-guided biopsies of these lymph nodes are not routinely performed by respiratory physicians. Recently, several studies have shown the benefits of incorporating routine neck US into the lung cancer diagnostic algorithm. The aim of this narrative review is to summarize the current literature with regards to the training strategies, requirements, and needs for trainees to become competent in performing neck US and US guided lymph node biopsies. Following the implementation a pre-determined search strategy, 11 studies were identified as suitable and included in this review. Based on the included studies, we created a guideline for both trainers and trainees whose goal is to achieve competency in both neck US examination and US guided lymph node biopsy.

## Introduction

The Global Cancer Observatory (GLOBOCAN) 2020 reported lung cancer to be the leading cause of cancer related deaths with approximately 1.8 million patient deaths in 2020, which equates to 18% of the total cancer deaths [1]. While breast cancer is the most frequently occurring cancer in women, lung cancer is the most diagnosed cancer in men as well as the highest cause of cancer related death in men and the second highest in women [1]. A 2025 report by the American Cancer Society reported that in the United States, lung cancer has one of the least favourable 5-year survival rate at 27% [2]. In addition there is a global increase in lung cancer incidence especially in developing countries and therefore there is an increasing need for easily accessible diagnostic procedures [3]. Furthermore, the emergence of immunotherapy and other directed-therapies for the treatment of lung cancer has increased the need to obtain accurate staging and tissue for molecular studies [4]. There is a linear increase in cervical lymph nodes involvement in lung cancer patients with TNM staging [5]. In patients with T4 staging, prevalence of pathological cervical lymph node may be present in over 30% of patients [6]. While patients with N1, N2, and N3 staging may have pathological cervical lymph nodes in approximately 20%, 30%, and 100% respectively as reported in a 2023 study [7]. More importantly, the involvement of this group of lymph nodes reflects either stage N3 or M1b which could be easily missed if cervical lymph nodes are not examined leading to poorer prognosis due to understaging [8].

Neck US and cervical lymph node biopsies are infrequently performed by respiratory physicians. This is likely because neck US and biopsies are not incorporated into the standard respiratory training curriculum in most training centres. A number of small studies that have examined the incorporation of routine neck US and cervical lymph node biopsies performed by respiratory physicians as part of the lung cancer algorithm and have reported it to be feasible, on top of other benefits such as shorter time to diagnosis and the avoidance of more invasive procedures [7, 9]. Presently, there are no training guidelines or recommendations for respiratory trainees on performing neck US and cervical lymph node biopsies. The purpose of this review is to summarize the current available evidence with regards to the training strategies, requirements, and needs for trainees to obtain competency in performing neck US and cervical lymph node biopsies. This paper is focussed on respiratory trainees as our main target audience, but could, in theory, include otolaryngologist and radiology trainees.

## Method

This review is constructed in the form of a narrative review. Search terms were determined between two authors LT and DB before it was carried out on MEDLINE/PubMed. No time limit was set as we expected limited literature to be available on this topic. The following search strings were used:

- ((neck US{MeSH Terms}) OR (US guided cervical lymph node biopsy{MeSH Terms})) AND
- (training{MeSH Terms})
- ((neck US{MeSH Terms}) AND (cervical lymph node biopsy{MeSH Terms})) AND (training{MeSH Terms})
- ((neck US{MeSH Terms}) AND (cervical lymph node{MeSH Terms})) AND (training{MeSH Terms})
- ((neck US{MeSH Terms}) AND (cervical lymph node biopsy{MeSH Terms})) AND (training{MeSH Terms})
- ((neck US{MeSH Terms}) AND (lymph node biopsy{MeSH Terms})) AND (training{MeSH Terms})
- ((neck US{MeSH Terms}) AND (aspiration biopsy{MeSH Terms})) AND (training{MeSH Terms})

Search results were imported into Rayyan for screening. Rayyan was used to organize studies for screening and to identify potential duplicates which were then manually reviewed by the authors. Abstract screening was done manually by authors through Rayyan. Full text screening was done through PubMed using full-text links to journals. Studies involving medical trainees or physicians/surgeons in the context of training were included, where else studies not involving medical trainees or physicians/surgeons not in the context of training were excluded. Studies involving neck US +/- cervical lymph biopsy were included in this study. Neck US involving thyroid nodules and biopsy of thyroid nodules were included as well following discussion between the authors as expected literature pertaining to lymph nodes alone would be scarce. Furthermore, the authors felt that the same principals in training can be applied to cervical lymph nodes. Studies involving all other forms of US and other types of biopsies were excluded. The bibliography of included studies was examined as well. Only articles in the English language were included.

## Results

**Fig. 1:**
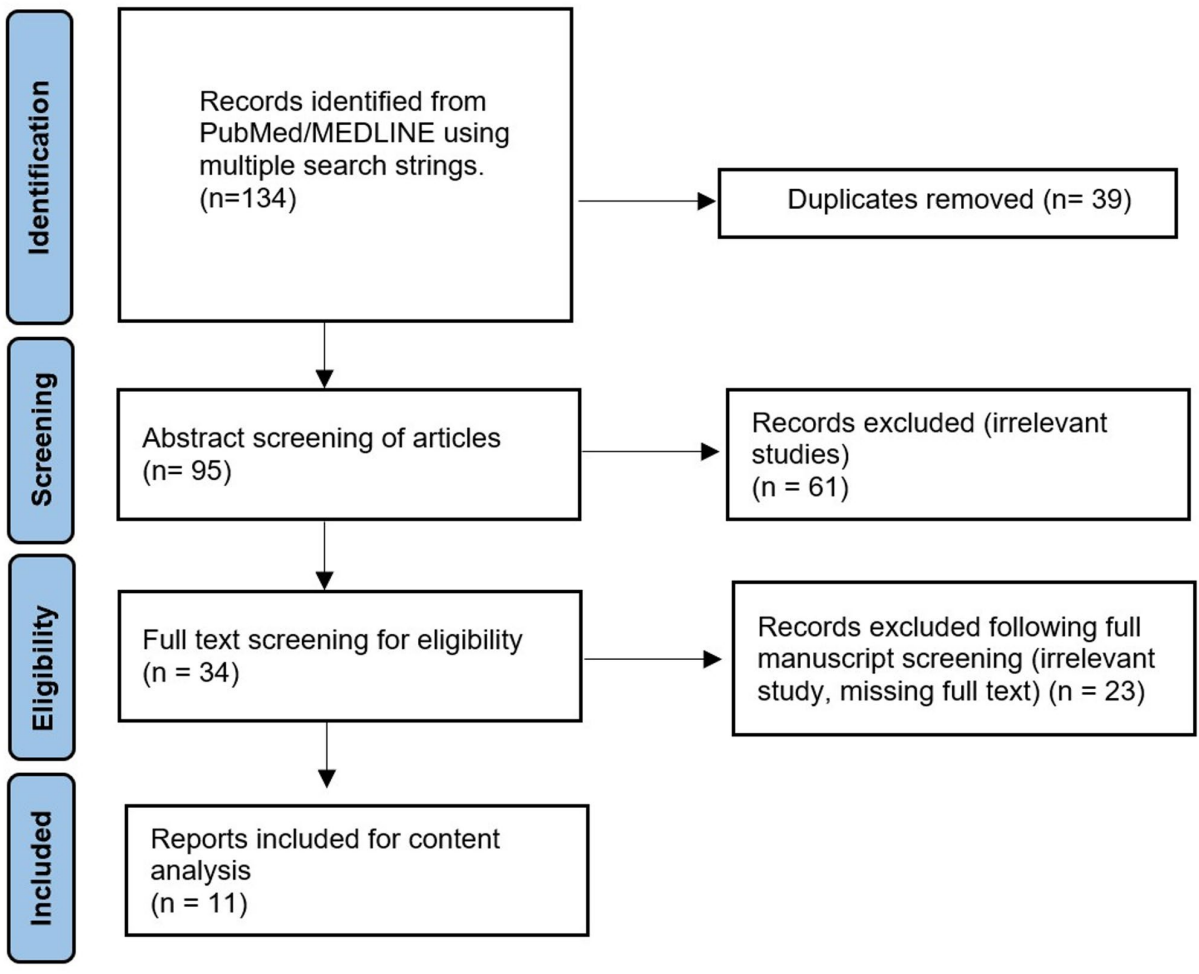
PRISMA flow diagram of articles screened

A total of 134 records were initially retrieved. Following removal of duplicates, manuscripts that were missing full text, and irrelevant articles, a total number of 11 articles were included in this manuscript (Figure 1). It was noted by authors that 3 main themes were identified, i.e. training in head and neck US, training in US guided biopsy, and simulation training.

### Achieving competency in head and neck US

The first step in achieving an adequate skill set in neck biopsies is to become competent in performing a thorough head and neck US examination. Basic theoretical knowledge is required prior to any US training and should include physics and instrumentation, US techniques, and administration which are skills often already possessed by most respiratory trainees from pleural US training as some of these skills are transferable [10]. The European Federation of Societies for US in Medicine and Biology (EFSUMB) have described their training requirements for head and neck US [10]. The overall principals for a high-quality head and neck US examination involves applying a systematic examination approach, good US technique, and focused reporting [10]. The importance of structured reporting is supported by a 2019 study investigated the impact of structured reporting on developing head and neck US skills. The authors reported that structured reporting significantly received better ratings in terms of report completeness and trainees greatly benefited from as the structured report help lead less skilled examiners through the examination more effectively [11].

A 2024 study investigated the effectiveness of Peyton’s four-step method combined with a flipped classroom approach in teaching US zoning of the thyroid and cervical lymph nodes for standardized residency training yielded compelling results [12]. Peyton’s four-step method is a learning model that consists of 4 steps. The first step is demonstration where the trainer performs the procedure at normal pace, followed by deconstruction with the trainer talking the trainee through each step while performing the procedure again [12, 13]. Step 3 is comprehension where trainer performs the procedure for the third time with sub steps described to them by the trainee [12, 13]. The final step is performance where the trainee performs the procedure on their own [12, 13]. The investigators observed that trainees who underwent Peyton’s four-step method had higher scores in operational skills and clinical case analysis compared to the control group. Furthermore, the intervention group were statistically significantly more likely to show improvement in learning interest and reduced exam pressure [12]. This study was a multicentred randomized controlled trial hence provided strong evidence for causal relationships, although, sample size was relatively small.

One study by Badran K et al. evaluated a one-to-one training model of neck US for an otolaryngologist trainee whom had no previous specialist imaging experience, in acquiring neck US skills. Authors reported it to be a feasible and acceptable approach when done under close supervision and input from an experienced radiologist [14]. This, however, was preceded by an induction process that included a 2-day practical US course, observing a number of neck US examinations performed by an experienced sonographer, informal tutorials (physics, instrumentation, and US anatomy of the neck), and early practical experience on normal volunteers [14]. This study was insightful given it reported the experience of a trainee with no prior experience in sonography. However, the study is limited by the fact that it involved a single trainee with no other comparators.

Assessing the competency of a trainee following US training is also a crucial component of the learning process. Todsen et al. examined the validity of using a competency-based assessment tool (Objective Structured Assessment of US Skills - OSAUS) in a 2017 study. The authors found that by using video-recorded US performance and the OSAUS scale [15], examiners were able to accurately differentiate experience sonographers from novice operators [15, 16]. This overall supports the use of competency-based assessment tools as part of the training assessment in achieving competency in head and neck US. The main strength of this study is participants in this study had different level of experiences which provided heterogeneity. The study was blinded which increased the validity of the findings. Unfortunately, the study sample size was small.

### Achieving competency in US guided cervical lymph node biopsy

The EFSUMB and the Royal College of Radiologists guidelines describes 3 levels of competency in head and neck US: Level 1 involves having the skills and knowledge to perform essential head and neck US examinations independently, Level 2 having the skills to perform US guided procedures, and Level 3 requires a more advanced level of training and practice with advance knowledge of US technologies and engagement in education or research [10]. Hence, it is recommended by the EFSUMB and the Royal College of Radiologists that clinicians who perform US-guided interventions should possess Level 1 and Level 2 competencies.

In 2020, Ahmed M et al. examined the effects of a 2-phase training model in training a respiratory physician to perform US-guided neck lymph node aspiration in patients with suspected lung cancer [9]. The 1st phase of the training involved the delegate being trained in the procedure consisting of supervised neck US examination, attendance in neck lumps clinic led by radiology and otolaryngologist surgeons, observership in the pathology department, use of simulation tools, and supervised neck node sampling. Phase 2 consisted of the trainee performing all procedures independently [9]. Following this 2-phase training method, the investigators concluded that it was a feasible mode of training and the trainee sustained an acceptable adequacy rate of sampling when compared to the standard set by the Royal College of Radiologists [9]. It is worth nothing however, that the trainee had possessed Level 1 Royal College of Radiologists prior to the commencement of the study. A 2023 study described a 5 stage process of training over a 6-month period that consisted of observation, performing neck US screening, assisting with biopsy procedures, performing biopsies with assistance, and performing procedures under observation [7]. The trainee was deemed as independent after being able to performed 5 directly supervised procedures without assistance from the trainer [7]. These two studies are highly applicable to our main target audience (respiratory trainees) given that they report the learning experience and outcomes of trainees with respiratory background. Both studies however, only involved a single trainee each with a certain baseline level of skill set hence may not be reflective of all respiratory trainees. Another study examined the effects of training two advance practice providers (APP) whom had significant pre-existing US guided procedure experience and familiarity with US studies of the neck in US-guided FNA procedures for superficial neck masses. Following a short period of observation and subsequent procedures performed under direct supervision, the trainees were able to independently perform cases with adequacy rate comparable to that of physicians [17]. At the end of this study, the authors compared the outcomes of the two trainees with that of an experience interventionalist which provided a valid measure of outcome. However, the study included only 2 trainees whom both have similar level of experiences in US-guided procedures prior to the commencement of the study.

A 2015 study was conducted by an otolaryngologist surgeon performing thyroid nodule biopsies examined the learning curve of this procedure. The authors reported an increasing cytology adequacy rate over time, from 72% in the first 100 cases to 78% for the following 100 cases and finally an adequacy rate of 85% for the last 100 cases [18]. The authors concluded that a learning process exists as demonstrated by the incremental increase in adequacy rate over time. Reporting on the learning curve of a trainee in this procedure provided an interesting insight into the training process however this study was retrospective in nature hence likely has an increased risk of biases being present.

### The use of simulation training

While training towards competency in performing head and neck US examinations can be easily performed on real-life patients, the same may not be as easily applied for US guided interventions given that it is more invasive. Hence, some trainers may argue that simulation training may be a necessary transitional tool for trainees in achieving competency in US guided neck biopsies.

A 2024 study described a training method to simulate head and neck US-guided procedures by using a gelatine phantom model [19]. The investigators custom-built a rudimentary and inexpensive model using easily attainable objects to simulate anatomically accurate structures of the neck as a simulation tool for training in US guided neck biopsies. The investigators reported that the use of this gelatine model is an effective educational method for procedures such as fine needle aspiration (FNA) and core-needle biopsy (CNB) and participants were able to gain a thorough understanding of the procedure and acquire essential skills necessary for the procedure [19]. Furthermore, trainees reported a high level of satisfaction with the simulation course with higher scores seen in trainees without prior US experience [19]. This study was able to demonstrate that simulation training does not require high expenses given that authors were able to create a cheap model that can be easily replicated by readers. However, study numbers were low and not all participants responded to the final questionnaire set by the investigators. Another study investigated the use of a fresh cadaver model for US FNA of thyroid nodules and found it to be an effective simulation tool in improving students’ skills. There was a statistical improvement in performing FNA following a period of training [20]. Although this study lacked a control arm, results were encouraging given that all students had no prior US training which provided insight into the benefits of simulation training in trainees with no prior sonography experience.

The use of Augmented Reality (AR) is a relatively new concept in simulation training. A 2024 study explored the use of AR technology with head-mounted displays (HMD) for US guided neck interventions [21]. This crossover study compared the performance and cognitive load of trainees attempting accurate central venous catheter placement using an AR-HMD to display US images compared with standard US without visual overlay. The authors reported that AR technology improved trainee performance and reduced cognitive load during the procedure [21]. Cognitive load is the amount of mental resources required to successfully complete a task and to process information related to a task [22]. High cognitive load has been previously associated with reduced learning outcomes especially when it exceeds the trainee’s working memory capacity [23, 24]. Cognitive load was measured by the investigators using the National Aeronautics and Space Administration Task Load Index (NASA-TLX) tool. Higher cognitive load has been previously associated with increased procedural complications and decreased learning capacity [21, 25]. The authors used a highly validated tool to measure cognitive load which increased the validity of the results. AR is costly and most centres may not be able to afford it hence decreasing applicability of this study to a lot of institutions.

## Guidance for trainers and trainees

**Fig. 2:**
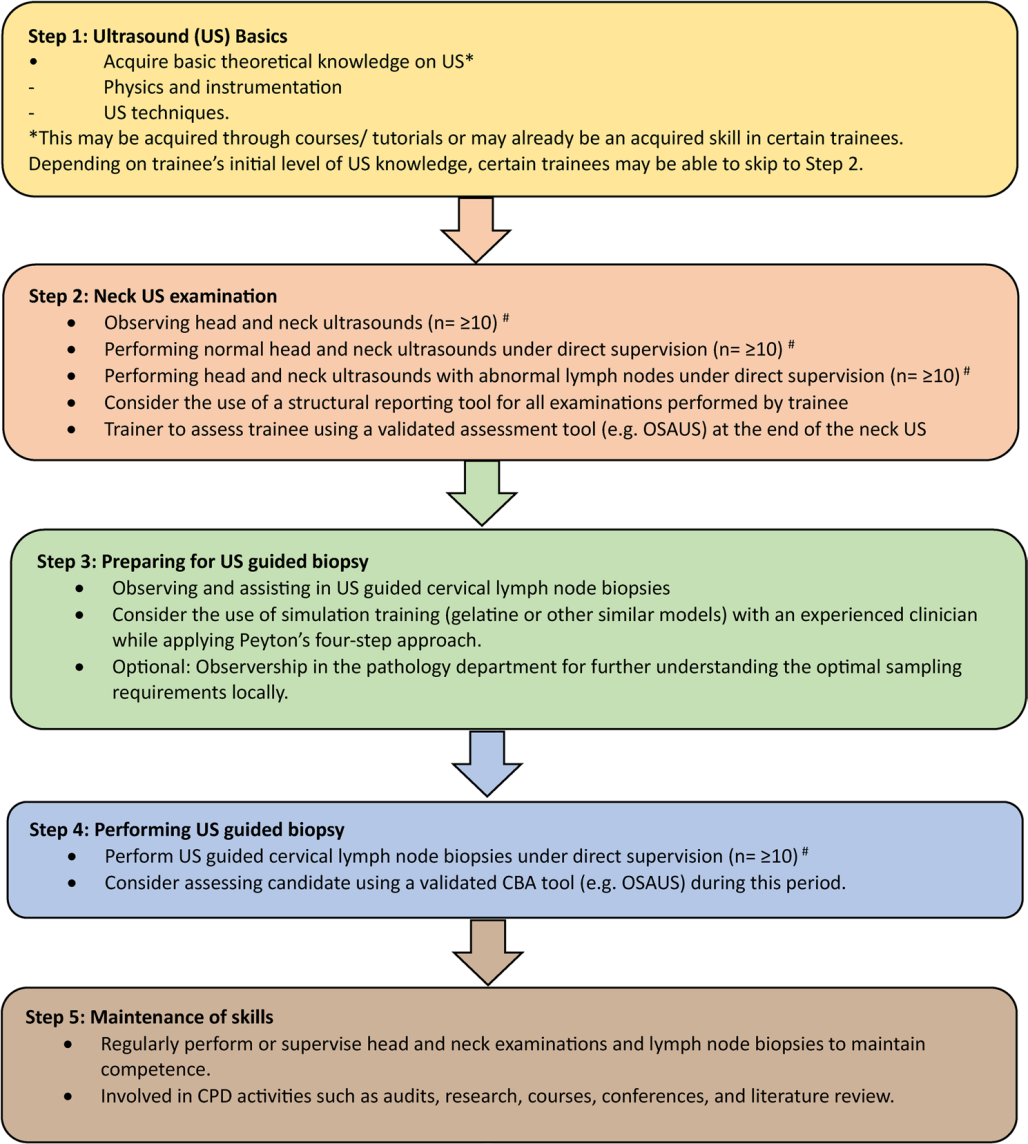
Diagram showing our recommendations for trainers and trainees in achieving competency in US guided cervical lymph node biopsies. ^#^ Numbers may vary across trainees depending on base level of experience (n = ≥ 10 is based on expert opinion). CBA = Competency Based Assessment; CPD = Continuous Personal Development; OSAUS = Objective Structured Assessment US Skill Scale; US = Ultrasound

We have summarized the current existing literature on training in neck US examination and US guided neck biopsy in Table 1. Based on the combination of current available evidence and the collective experiences of the authors in training and performing the procedure, we have created a diagrammatic process of our recommendations for trainers and trainees who wish to train or be trained at head and neck US examinations and US guided biopsies of cervical lymph nodes **(**Fig. 2**).**

**Table 1:** Summary of relevant study in training for neck US examination and US-guided biopsy of lymph nodes. APP = Advance practice Providers; AR = Augmented Reality; CBA = Competency based assessment; FCM = Fresh cadaver Model; FNA = Fine needle Aspiration; OSAUS = Objective structured assessment of ultrasound Skills; US = ultrasound. *Evidence appraised using the GRADE tool [26]

Authors	Year	Study design	Theme/topic	Study aim	Main relevant findings	Study limitations	Evidence level*
Wang J et al. [12]	2024	Randomized controlled trial	Training method in US examination of thyroid and cervical lymph node	Investigate whether combining flipped classroom approach with Peyton’s four-step method enhances teaching effectiveness in neck US examination	- Intervention group had higher scores in skill operation and clinical case analysis - Greater learning interest in intervention group	- The study only consisted of 2 centres. - Small sample size (*n* = 66)	Moderate
Cheng PC et al. [19]	2024	Prospective observational study	Gelatine model in neck US guided procedures	Evaluate the efficacy of a training program using a gelatine phantom to practice US-guided procedures.	- High satisfaction from trainees when gelatine model was incorporated to training. - Gelatine models benefited trainees with no prior US experience the most.	- Small sample size (*n* = 44) - < 100% response rate to questionnaire	Low
Liao SC et al. [21]	2024	Randomized crossover study	AR in US-guided neck interventions	Determine the effectiveness of AR in enhancing physician training for US-guided interventions using AR visual overlays	- AR reduced time required to perform procedure - Cognitive load was reduced in trainees who utilized AR.	- Small sample size (*n* = 47) - All participants had no prior experience in using AR	Moderate
El-Shaarawy B et al. [7]	2023	Prospective observational study	Respiratory physician trained in performing US-guided FNA of cervical lymph node in lung cancer	Clinical utility of US guided cervical lymph node biopsies in patients with lung cancer by a respiratory physician following a period of training.	- Trainee underwent 5 stages of training i.e. observation, performing neck US screens, assisting with biopsy procedures, performing biopsies with assistance, and performing procedures under observation.	- Study reports the experience of a single trainee	Very low
Paez SN et al. [17]	2022	Prospective observational study	Performance of APP in US-guided FNA of thyroid nodules and neck masses following training.	Compare the adequacy rate of APP in US-guided FNA neck procedures compared to physicians following training	- APP with prior experience in other US-guided procedures can achieve comparable results in adequacy rates to physicians following a short period of observation and one-on-one supervised sessions.	- Observational study of only 2 trainees with similar baseline experience.	Very low
Ahmed M et al. [9]	2020	Prospective observational pilot study	The learning process of neck US examination and lymph node biopsy	Assess the feasibility of training a respiratory physician to perform neck US examination and needle sampling of cervical lymph nodes	- The trainee who had a background of Level 1 training, following one-on-one sessions (with supplementary sessions) with an experienced operator, achieved good adequacy rates in sampling.	- Study reports the experience of a single trainee	Very low
Ernst BP et al. [11]	2019	Randomized controlled trial	Structured reporting in neck US examination.	Evaluate the impact of structured reporting of head and neck US examinations on the learning process	- Better quality and time to complete report with structured reporting may benefit the learning process of trainees.	- Single centre study - Participants recruited from a course (might not be representative general population)	Low
Todsen T et al. [16]	2018	Prospective experimental study	Using CBA tool in head and neck US as part of training	Evaluate the validity of OSAUS as a CBA tool.	- OSAUS had good reliability in discriminating experienced vs. notice operator hence useful as a learning tool - Strong co-relation between OSAUS score and diagnostic accuracy	- Small sample size (*n* = 17)	Low
McCrary HC et al. [20]	2017	Prospective observational study	FCM as simulation tool in US-guided FNA of thyroid nodules	Determine if a FCM for the instruction of US-guided FNA of thyroid nodules is a practical method for instruction.	- Using a FCM is useful in increasing cognitive knowledge gain in students when comparing pre- and post-instruction skills assessment.	- Observation study of a small sample size (*n* = 17)	Low
Fernandes VT et al. [18]	2016	Retrospective observational study	Learning curve in US guided FNA of thyroid nodules	Report on a learning curve and impact of head and neck surgical trainees on adequacy rates.	- Adequacy rates increase over time/cases indicating a learning process with excellent rates achieved after 300 cases.	- Retrospective study (increase in bias)	Very low
Badran K et al. [14]	2014	Prospective observational study	Outcomes and challenges in learning the technique in US neck examination	To assess the feasibility and accuracy of otolaryngologist-performed US in evaluating head and neck pathology	- One-to-one training model for neck US for an otolaryngologist trainee with no previous experience is a feasible method to achieve competency.	- Study reports the experience of a single trainee	Very low

## Discussion and conclusion

While most respiratory physicians are trained at performing pleural US, by and large, respiratory trainees would be unfamiliar with performing neck US. Given that most respiratory departments should have relatively easy access to an US machine for pleural imaging and procedures, the incorporation of a service providing neck US would be an easy addition for most services. Furthermore, most US machines would be equipped with both curvilinear and linear probes which allows neck procedures to be added without incurring additional cost to a department.

The existing literature on the training strategies for US neck examination and US-guided cervical lymph node biopsy is currently limited even after including literature that pertains to thyroid nodules and other neck masses. While including thyroid nodules allowed us to examine more literature, we acknowledge that there are drawbacks to this. Thyroid nodules are mainly in the realm of otolaryngology and interventional radiology, hence there would likely be major differences in baseline knowledge of the neck anatomy and sonography skills between trainees of different subspecialties. Furthermore, while the principles of thyroid nodule biopsies are similar to that of cervical lymph nodes, the anatomical region for the latter is more substantial hence techniques involved in biopsy of thyroid nodules may not be fully transferable to certain regions in the neck e.g. supraclavicular region and submandibular region.

Most of the included studies were small hence there is an increased risk of selection bias and survivor bias. Furthermore, as significant proportion of included studies were observational which increases the likelihood of observational bias. Given that our recommended guidelines are partly based on authors’ experiences (all authors bar MB are specialized in the field of respiratory medicine), we acknowledge that our guidelines may not be fully applicable to some cohort of trainees, especially non-respiratory trainees. In particular, the level of baseline knowledge and existing skill-set in sonography will vary across different specialities as previously mentioned hence training needs and requirements may differ.

A major theme that was covered by this review is simulation training given its increasing usage in the last decade. Simulation training has been shown to be effective in other forms of US-guided biopsies such as renal biopsy, soft tissue biopsy, and prostatic biopsy [27–29]. A previous study that investigated the use of a simple abdominal phantom device for US guided biopsy of small lesions observed that radiology residents who had undergone simulation training had reduced time in performing the biopsy and required less needle punctures or adjustments to satisfactorily complete the procedure [30]. Although evidence thus far for neck biopsies and other regions support the use of simulation training, it should not replace real-life hands-on experience as there are factors that will not be reproducible in simulation training such as patient’s tolerance to procedure and anatomical variants between patients. Furthermore, the neck region is anatomically highly complex in comparison to many other anatomical regions therefore trainers/trainees should not assume that procedural skill sets from a different anatomical area is completely transferable. Arguably, high-fidelity simulation could possibly reduce the learning curve when transferring skills from one form of biopsy to another but it can be expensive and may not be affordable by most training centres. Nevertheless, trainers and trainees alike should not be discouraged with low fidelity models as the use of simple simulation tools can be beneficial in the learning process. Interestingly, a randomized crossover study examining low versus high-fidelity simulation in basic laparoscopic skills training found that trainees in the low fidelity-simulation group had a higher mean crossover score suggesting that training in low fidelity models allowed better transfer of skills to new settings [31].

Assessing trainees following instruction is also an important component in any learning process. The use of structured assessment tools such as the OSAUS have been long used in assessing and training candidates in various specialities in medicine [32, 33]. Incorporating structured assessment tools would allow trainers to objectively assess trainees in an efficient way and provide constructive feedback. Repeated assessment throughout training period will also provide trainees with insight into how their skills have developed over time allowing for reflection, an important but often neglected component in learning [34]. Trainers should also encourage trainees to constantly reflect on their experiences as it encourages life-long learning.

In conclusion, the current existing literature on training strategies for US neck examination and US-guided cervical lymph node biopsy is currently limited and is mostly of low quality. Reassuringly, literature has shown that competency in US neck examination and cervical lymph node biopsy can be achieved by trainees even without prior experience in US skills if adherence is maintained to proper training methods. A basic knowledge in US techniques is a fundamental first step in this learning process. This is followed by supervision from an experienced operator along with the use of a structured reporting system and CBA tools will greatly enhance the learning process. Simulation tools can be extremely helpful to trainees when learning US-guided cervical lymph node biopsy especially for trainees who have no prior experience in performing US-guided procedures.

The maintenance of skills is as important as the learning process, hence, once competency is achieved, in addition to regularly performing the procedure, trainees are encouraged to be actively involved in continuous personal development activities that will further improve their skills and knowledge. Further high-quality studies are needed to compare the type of training model that is ideal in the learning process of US neck examination and US-guided cervical lymph node biopsies.

## Data Availability

No new data was generated from this study.

## Abbreviations

APP: Advance Practice Provider
AR: Augmented Reality
CBA: Competency Based Assessment
CPD: Continuous Personal Development
EFSUMB: European Federation of Societies for Ultrasound in Medicine and Biology
FCM: Fresh Cadaver Model
FNA: Fine Needle Aspiration
GLOBOCAN: Global Cancer Observatory
HMD: Head-mounted Display
IASLC: International Association for the Study of Lung Cancer
NASA-TLX: National Aeronautics and Space Administration Task Load Index
OSAUS: Objective Structured Assessment Ultrasound Skill Scale
US: Ultrasound
